# Laparoscopic resection of a neuroendocrine tumor that almost fully replaced tailgut cysts: a case report

**DOI:** 10.1186/s40792-020-01044-z

**Published:** 2020-10-17

**Authors:** Keita Kodera, Seiichiro Eto, Nei Fukasawa, Wataru Kai, Tomo Matsumoto, Tsuyoshi Hirabayashi, Hidejiro Kawahara, Nobuo Omura

**Affiliations:** 1 Department of Surgery, National Hospital Organization Nishisaitama‐Chuo National Hospital, Wakasa 2‐1671,, Tokorozawa‐shi, Saitama 359‐1151 Japan; 2 Department of Pathology, The Jikei University School of Medicine, 3-19-18 Nishi-Shimbashi, Minato-ku, Tokyo 105-0003 Japan

**Keywords:** Neuroendocrine tumor, Tailgut cyst, Presacral mass

## Abstract

**Background:**

Neuroendocrine tumors (NETs) originate from neuroendocrine cells, which are found throughout the body. NETs occur principally in the gastrointestinal tract (approximately 65%) and bronchopulmonary tract (approximately 25%) but rarely occur in the presacral space. Aside from primary and metastatic lesions, there have been reports of NETs occurring in the presacral space arising from tailgut cysts, teratomas, and imperforate anus. We herein report a rare case of laparoscopic resection of a NET in the presacral space, which almost fully replaced tailgut cysts.

**Case presentation:**

A 68-year-old woman was referred to our hospital for surgery of a right inguinal hernia, but preoperative computed tomography revealed an asymptomatic 43-mm mass in the presacral space. Magnetic resonance imaging showed a multilocular solid mass with clear boundaries and a slightly high signal intensity on T1- and T2-weighted images. Positron emission tomography showed 18F-fluorodeoxyglucose uptake. Thus, we suspected a malignant tumor and performed laparoscopic resection to obtain a definitive diagnosis. Macroscopically, the tumor was 43 mm in size with clear boundaries, and the cut surface was a gray-white solid component. Histopathological findings revealed that the tumor was composed of relatively uniform cells with fine chromatin, with round to oval nuclei arranged in solid, trabecular, or rosette-like growth patterns. Small cysts lined with stratified squamous epithelium and columnar epithelium were observed along with solid components of the tumor, which is a feature of tailgut cysts. Therefore, the final diagnosis was NET Grade 1 arising from tailgut cysts. No recurrence was observed within 1 year after surgery.

**Conclusions:**

We performed en bloc laparoscopic resection of a NET arising from tailgut cysts in the presacral space without injury. In cases of a solid lesion in the presacral space, not only the primary disease but also the pathological condition with tissue transformation and replacement should be considered, as in this case.

## Background

Neuroendocrine tumors (NETs) originate from neuroendocrine cells, which are found throughout the body. NETs occur principally in the gastrointestinal tract (approximately 65%) and bronchopulmonary tract (approximately 25%) but rarely occur in the presacral space [1]. Aside from primary and metastatic lesions, there have been reports of NETs occurring in the presacral space arising from tailgut cysts, teratomas, and imperforate anus [2]. We herein report a rare case of laparoscopic resection of a NET in the presacral space, which almost fully replaced tailgut cysts.

## Case presentation

**Fig. 1:**
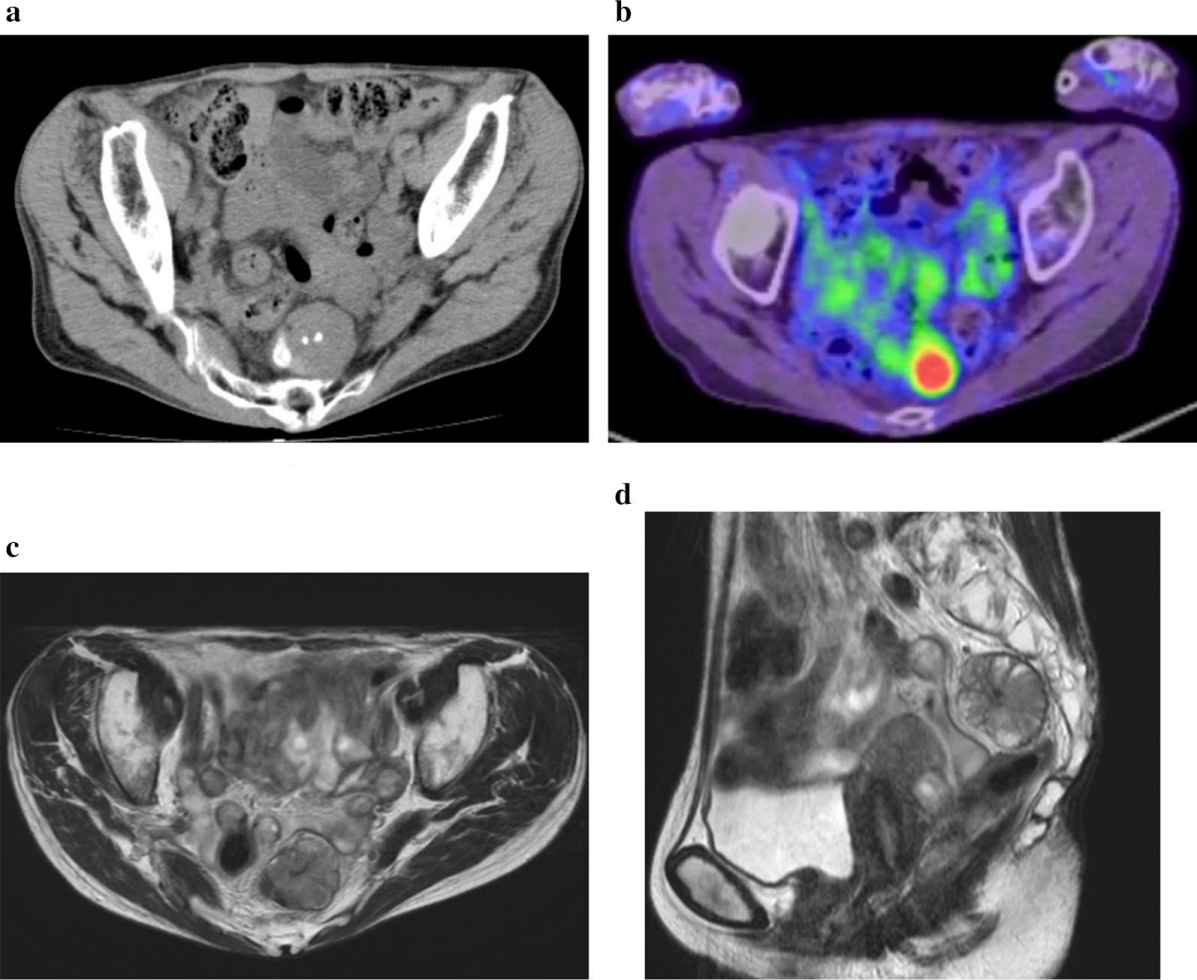
**a** CT revealed a 43 mm-sized mass with clear calcification on the ventral side of S3. CT, computed tomography. **b** FDG PET/CT showed strong accumulation with an SUVmax of 10.3 in the tumor. FDG PET/CT, 18F-fluorodeoxyglucose positron emission tomography/computed tomography; SUVmax, maximum standardized uptake value. **c**, **d** MRI revealed a multilocular 43 mm-sized mass with well-defined lobules and septa at the same site. There was no sacral invasion. T2-weighted images showed a slightly high signal intensity. MRI, magnetic resonance imaging

In 2018, a 68-year-old woman was referred to our hospital for surgery for a right inguinal hernia. Preoperative computed tomography (CT) revealed an asymptomatic mass 43 mm in size in the presacral space. Her medical history included Sjogren's syndrome, renal tubular acidosis, and Hashimoto's disease. Although the details were unknown, she had an allergy to intravascular contrast agents. None of her family had a clear history of cancer. Hematological examination showed no elevations in tumor markers or inflammation. No abnormality was observed on upper and lower gastrointestinal endoscopy. CT revealed a 43 mm-sized mass with clear calcification on the ventral side of S3 (Fig. 1a). Magnetic resonance imaging revealed a multilocular 43 mm-sized mass with well-defined lobules and septa at the same site, which was observed as slightly high signal intensity on T1 and T2 images, high signal intensity on diffusion-weighted images, and low signal intensity on the apparent diffusion coefficient map. Sacral invasion by the tumor was not observed on imaging (Fig. 1b, c). 18F-fluorodeoxyglucose positron emission tomography/CT showed strong accumulation with a maximum standardized uptake value of 10.3 in the tumor (Fig. 1d). Thus, we suspected a malignant tumor in the presacral space, such as a malignant schwannoma, metastatic malignant tumor, extragastrointestinal stromal tumor, solitary fibrous tumor, and malignant transformation of a teratoma or tailgut cyst.

**Fig. 2:**
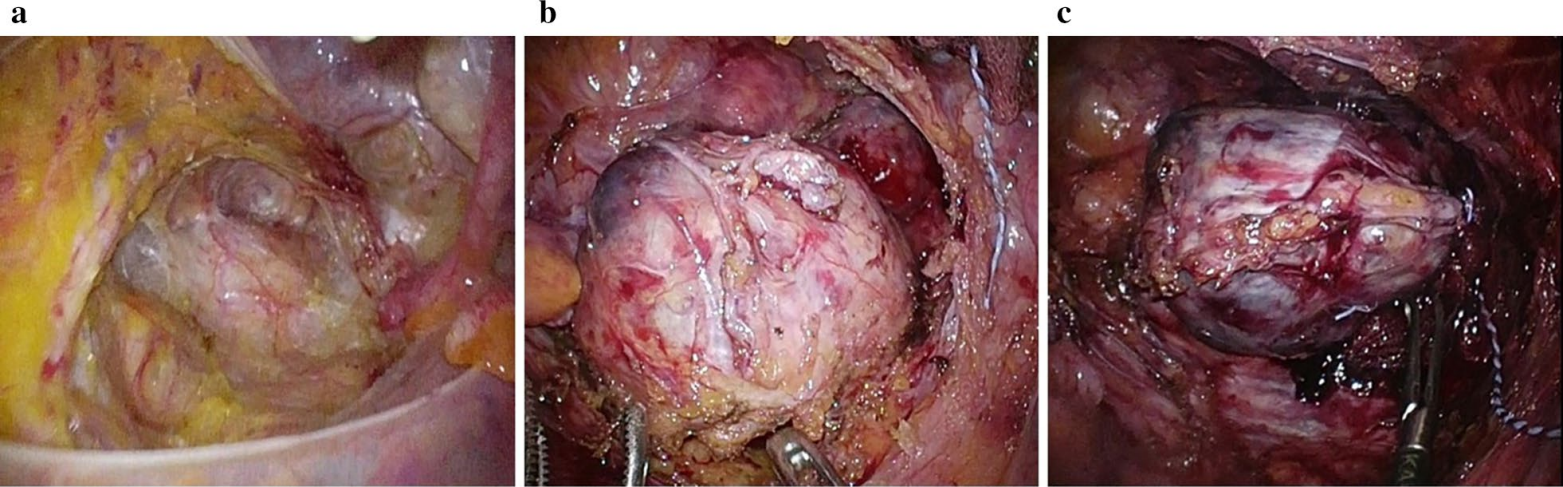
**a** The retroperitoneum was dissected caudally from the promontrium using a medial approach, and the rectal mesentery was dissected. After mesenteric mobilization, a well-defined mass was revealed in the presacral space. **b** Detachment was performed, except for the anterior aspect of the sacrum. **c** Dorsal detachment in contact with the front of the sacrum was performed last. The tumor was resected en bloc without any exposure

We performed laparoscopic surgery to obtain a definitive diagnosis. After administering general anesthesia, the patient was placed in the lithotomy position and underwent laparoscopic surgery using 5 ports. As in rectal surgery, the retroperitoneum was dissected caudally from the promontrium using a medial approach, and the rectal mesentery was dissected. After mesenteric mobilization, a well-defined mass was observed in the presacral space (Fig. 2a). The rectum was completely divided from the tumor, and the rectum and uterus were suspended to obtain a good visual field. The tumor located dorsal side of pre-hypogastric nerve facia and did not show clear communication with the hypogastric nerve and pelvic nerve plexus. The main feeding vessels of the tumor were the branch of the median sacral vein and some branches of the superior rectal artery. The tumor was rolled using gauze to prevent capsular injury and then detached. The inflowing blood vessel was carefully sealed using laparoscopic coagulation shears. The dorsal detachment in contact with the front of the sacrum, where bleeding was expected, was performed last (Fig. 2b). The tumor was resected en bloc without exposure (Fig. 2c). Although a small amount of bleeding was observed in the anterior sacrum, hemostasis was achieved by compression and cauterization, using gauzes, an absorbable haemostats, a coagulation and spray mode of the electrocautery and an argon beam coagulator. The operation time was 296 min, and the blood loss volume was 314 mL. The postoperative course was uneventful, and the patient was discharged on the 7th postoperative day. The patient is currently undergoing outpatient follow-up for 1 year after the surgery, with no recurrence.

**Fig. 3:**
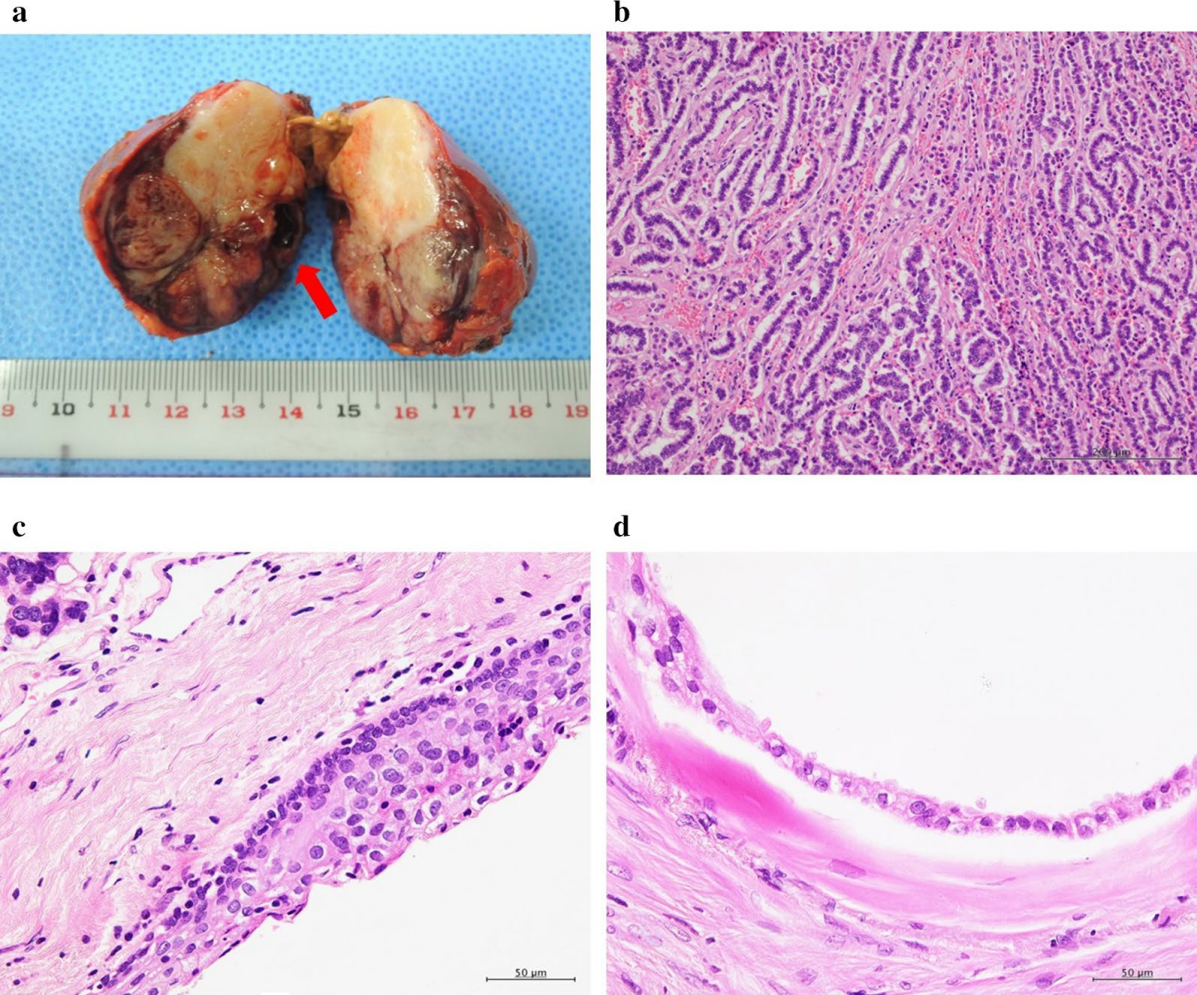
**a** The resected specimen was covered with a fibrous capsule and had a well-defined mass. The cut surface was a gray-white solid component, and small cysts were found in the periphery (indicated by an arrow). **b** The tumor was composed of relatively uniform cells with fine chromatin, which had round to oval nuclei arranged in solid, trabecular, or rosette-like growth patterns (× 100 magnification). **c** Cysts found in the periphery were lined with stratified squamous epithelium (× 400 magnification). **d** Multiple small cysts were found within the solid component and lined with columnar epithelium (× 400 magnification)

The macroscopic findings of the resected specimen were covered with a fibrous capsule and had a well-defined mass. The cut surface was a gray-white solid component, and a small cyst was found in the periphery (Fig. 3a). Histopathologically, the tumor was composed of relatively uniform cells with fine chromatin, with round to oval nuclei arranged in solid, trabecular, or rosette-like growth patterns. On immunostaining, the tumor cells tested diffusely positive for synaptophysin and cluster of differentiation-56, somatostatin receptor subtype 2a (SSTR2a) and pancreatic polypeptide, and focally positive for progesterone receptor. No positivity for Chromogranin A, p53, estrogen receptor, gastrin, serotonin, somatostatin, CDX2 and TTF1 was observed. The Ki-67 (MIB-1) labeling index was less than 2%. Thus, she was diagnosed with a NET (Grade 1). The cysts found in the periphery were lined with stratified squamous epithelium (Fig. 3c) and contained flocculent eosinophilic material with focal calcification, which was considered to be necrotic tissue. Multiple small cysts were found within the solid component and lined with columnar epithelium (Fig. 3d). No components other than those derived from the ectoderm were found in the tumor. The presence of cysts lined by multiple epithelia was consistent with the characteristics of tailgut cysts, and it was considered that the NET originated from tailgut cysts and replaced almost all of them.

## Discussion

The presacral/retrorectal space is bordered on the ventral side by the rectum, on the cranial side by the peritoneal reflection of the rectosigmoid colon, on the caudal side by the rectosacral/Waldeyer’s fascia, on the dorsal side by the presacral fascia, and laterally by the iliac vessels and ureters. In this space, a variety of congenital and acquired tumors can occur, classified as congenital, neurogenic, osseous, inflammatory, or miscellaneous [3]. Presacral tumors may cause symptoms such as constipation, defecation, urination disorders, and lower abdominal pain as they grow. However, it is not uncommon for them to be found incidentally without causing symptoms, as in this case [4]. The principle of treatment for tumors arising in the presacral space is surgery. Surgical resection is considered a treatment option performed to eliminate the risk of tumor growth, tumor-related symptoms, infections, and malignancy, even when the tumor appears to be benign. Imaging studies are crucial for preoperative diagnosis and surgical planning. The surgical plan involves defining the localization and extent of the tumor and determining the approach and whether combined resection of the surrounding organs is necessary [5]. With recent technical improvements, histological diagnosis by preoperative imaging-assisted needle biopsy has been reported to be useful in improving the accuracy of diagnoses and reducing complications [6]. However, biopsy is not necessarily recommended as a routine procedure, mainly because of the risk of seeding and infection and the fact that surgical treatment is often required regardless of the results of the preoperative diagnosis [7, 8]. Needle biopsy should be performed only when imaging findings suggest a preoperative diagnosis, which may be an indication for preoperative chemotherapy and may reduce surgical invasiveness or avoid surgery, such as Ewing sarcoma, osteosarcoma, lymphoma, and solitary fibrous tumors [9]. Therefore, the final diagnosis is determined by the pathology of the surgically resected specimen.

There are three types of approaches for presacral tumors: the anterior approach, posterior approach, and combination of both. The anterior approach indicates a transabdominal approach, which is a good indication for tumors cranial to S3 without sacral invasion, providing a good surgical view of the pelvic vessels and ureters. The posterior approach implies a transsacral or transanal approach with good nerve visibility and is an indication for tumors located caudal to S3, especially in cases requiring caudal resection. The combined anterior and posterior approach is applied in cases of tumors around S3 with large tumor diameter and invasion of the sacrum and pelvic side walls [4, 5]. The key to surgery for presacral tumors is to reduce bleeding from feeding vessels of the tumor and the presacral venous plexus. Preoperative selective catheter embolization to feeding vessels in a hyper vascularized tumor could reduce the blood loss [10]. On the other hand, there is no reliable preoperative method of preventing bleeding from the presacral venous plexus, despite the risk of uncontrolled bleeding [11]. Thus, intraoperative control of the bleeding is very important and the bleeding should be minimized using electrocautery, laparoscopic coagulation shears, argon beam coagulator, and topical hemostatic agents. Laparoscopic resection of the anterior sacral masses has been reported to provide a good intrapelvic surgical view, reducing bleeding and nerve damage. Nedelcu et al. presented the indications for laparoscopic surgery of presacral tumors suspected to be benign, located above S3, less than 6 cm, and without invasion of other organs or bones [12]. There are very few reports of laparoscopic surgery in cases of suspected malignancy. This may be caused by the risk of dissemination due to tumor damage and uncontrolled bleeding. However, laparoscopic resection is a very good indication for a well-defined marginalized solid malignancy, as in the presented case, as well as for benign disease. We pushed the rectum up to the ventral side, rendering a good surgical view of the pelvis, and then dissect the anterior surface of the sacrum at the end of the procedure because of the expected bleeding. The magnification of the laparoscopic surgery allowed for sensitive hemostasis during the presacral procedure, resulting in operation with an acceptable amount of blood loss. We believe that laparoscopic surgery should be performed aggressively even in cases of suspected malignancy, provided that preoperative imaging is sufficiently reviewed to determine the appropriate indication.

NETs originate from neuroendocrine cells, which are found throughout the body, and occur principally in the gastrointestinal tract (approximately 65%) and bronchopulmonary tract (approximately 25%). Occurrence in the presacral space is rare [1]. Aside from primary and metastatic lesions, there have been reports of NETs occurring in the presacral space arising from tailgut cysts, teratomas, and imperforate anus [2]. Tailgut cysts are congenital lesions that arise in the presacral space. They are thought to arise from the remnants of the hindgut during the embryonic period. Their histological features include various types of epithelium such as stratified squamous, transitive, mucinous, ciliated, and cubic mucous epithelium lining the cyst. Tailgut cysts are primarily benign, but in about 30% of cases, malignant transformation to NETs, adenocarcinoma, squamous cell carcinoma, endometrioid carcinoma, and sarcoma has been reported. Among them, conversion to NETs is the most common, and about 30 cases have been reported in English literature. Previous reports have shown that NETs arising from tailgut cysts are frequently found in women and are symptomatic [13–15]. The mechanism of this transformation has been suggested to involve estrogen and ghrelin, but the details are not known [2, 16]. The principle of treatment is resection of the primary tumor, which has been performed in all but one reported case. Because of the small number of cases, there is no definite postoperative treatment or follow-up method. It is common to treat and follow up patients with NETs that occur in other organs. Pathologically, the proportions of NETs and tailgut cysts in each lesion are variable. Therefore, the lesions may be solid, cystic, or a mixture of both [13, 15]. NETs arising from tailgut cysts were positive on immunostaining for SSTR2a, as well as primary NETs. Therefore, somatostatin receptor scintigraphy is useful in preoperative diagnosis [2, 17].

In our case, the presence of cysts lined by multiple epithelia was consistent with the characteristics of tailgut cysts, and it was thought that the NET arising from the tailgut cysts replaced almost all the cysts. On immunostaining, estrogen which has been reported to may be involved in the mechanism of the transformation were not detected. Since the Grade 1 NET was a low-grade tumor and the tumor was completely resected, the patient was followed up without postoperative treatment.

## Conclusion

We performed laparoscopic resection of a NET arising from tailgut cysts in the presacral space without tumor injury. In the case of a solid tumor in the presacral space, not only the primary disease but also the histological condition with tissue transformation and replacement should be considered, as in this case.

## Data Availability

Data sharing is not applicable to this article as no datasets were generated or analyzed during the current study.

## Abbreviations

CT: Computed tomography
NET: Neuroendocrine tumor
SSTR2a: Somatostatin receptor subtype 2a
